# On the Future of the Humanities: Interview With Professor Mircea Flonta, Epistemologist and Philosopher of Science

**DOI:** 10.5964/ejop.v11i2.999

**Published:** 2015-05-29

**Authors:** Mircea Flonta, Beatrice Popescu, Andrei Simionescu-Panait

**Affiliations:** a Faculty of Philosophy, University of Bucharest, Bucharest, Romania

**Beatrice Popescu and Andrei Simionescu-Panait (EJOP)**: The last decade has hosted a fast pace of publishing from your part. What was the project towards which your efforts were directed over the last three years? Is it editorial?

**Mircea Flonta**: Many do not like to talk about their plans, about what they intended to do but never did. I can say certain things. I watched the daughter of Serban Ţiţeica - who recently translated a work of Heisenberg and one of Bohr's - I watched a project related to the interpretation of quantum mechanics, of the Copenhagen School, plus the controversies that have erupted from it. All this concerns a subject that interests me: the philosophy of the researcher. I mean, there is a philosophy of philosophers, you know? The philosopher of science seeks a better understanding of science in general, or of a particular field of science, in terms of conceptual aspects, while the researcher has its own philosophy, but this faces the following question: what the scientific achievements of the present represent, especially of the fundamental theories, and which further direction of research these theories seem to indicate. In this regard, scientists can hold different views and, furthermore, despite discussions carried with perseverance for a long time, within which the prospects are better clarified, they do not get to an agreement. That is because they have different background representations of what a scientific description or a scientific explanation, for instance, would be. These things change even under the influence of scientific developments over time and sometimes even men of the same generation come to controversies and misunderstandings due to their background influence. It is an interesting thing, which has preoccupied me for a long time. In 1985, I wrote a book named “Philosophical presuppositions in science” *(„Presupoziții filosofice în știința exactă”)*, which refers to this type of discussions – I published another book that went unnoticed, as well – “Images of science” *(„Imagini ale științei”)* – on the same subject. So during the last year and something I was caught up in this. I am not finished yet; I will get back to it, to reach a conclusion.

[Contrib contnr1]There was another important preoccupation during the last year and a half. Now, if you ask, I cannot hide it. I wrote a text, with the volume of a book, of about two hundred and something pages, entitled “My path to philosophy” *(„Drumul meu spre filosofie”)*. I went to the Faculty of Philosophy between 1951 and 1955 and I have been at the university ever since, 60 years now, and my colleagues have prepared a volume of homage, a Festschrift. To continue the text, I wrote another about 100 pages of comments on the work of my colleagues. This is what I did. The text should be published in summer or autumn.

**EJOP**: Accomplishing works of this caliber requires a labor discipline that transpires especially in the process of writing. How does a day of work go and how do you outline the steps of preparing a philosophy volume?

**Mircea Flonta**: I retired in 2002. This means that my work is reduced and I have more time. Of course, as the years pass by, I do not have the same resources, but I have much more time at my disposal. I feel better if every morning I stay 4-5 hours and work on something. I can read as well, but always seeking something. I learned during a long time and going through hard enough lacks, that you can read well only if you already have certain representations about the problem in question, because only then can you show active reading and take what is important from the rest. If that derives satisfaction, it means that I am having a good day and I feel fine the rest of the time.

About preparing manuscripts, I learned this: it is very bad when you have time, and I am referring here to doctoral theses. If you have a manuscript and do not stay on it for a few weeks, you will just leave it. Turning to it, you can easily make improvements. Usually, I do not impose restrictive time limits and I allow myself this opportunity to get back to that work a second time, perhaps even a third time, and it allows me to construct a better text than that I would have initially constructed. You can produce better things this way.

**EJOP**: We go back to the period when you were a professor. Both writing and teaching require time and reinterpretation. What is the history of the two aspects and how did the two combine?

**Mircea Flonta**: They are closely related. I fully agree with a close colleague from Germany who told me when I retired that “it’s hard to imagine how to carry on with philosophy without students”, him being a very active author. I am talking about Herbert Schnädelbach. I agree, because the practice of philosophy as an exercise of thinking is stimulating that way. Many of the things people do at some level is based on reading some other significant or first-rate authors, stimulators. If you discuss with students, for example at the seminars, such texts, you have the opportunity to see some things that do not come to light if you read the text three times sitting alone in a room. It is very important. In addition, there is the need to clarify things. My mentors (e.g., Wittgenstein) are people who felt this need to practice and are willing to go beyond clarification. For this, working with students helps a lot.

[Contrib contnr2]On the other hand, there is something else. If you are a teacher, you must have a broader thematic scope, as opposed to someone who is employed as a researcher. There are those institutes of the Academy, the philosophy and the psychology one at Constantin Radulescu Motru. The researcher has a work to plan, chooses to participate in collective works with a specific theme, and focuses all the lectures on the topic. It is never constrained by its professional duties to encompass a much wider scope, while a teacher should have a culture in specialization. I must have, at least in the philosophy of knowledge, the philosophy of science, etc., the obligation of reading great classic and contemporary works. During the last two decades, we insisted on master and doctoral seminars, and this is how I proceed: hold an introductory statement for the theme, present a list of texts, and then discuss them all, each at a time and this is not just for the benefit of the students. Moreover, I always take advantage of this - and feel the thematic horizon constantly remodelling. Therefore, I think the teaching profession and philosophical practice are closely linked.

**EJOP**: How was the philosophical atmosphere during doctorate, how did the meeting with Piaget go and what impact did a change of air have for you?

**Mircea Flonta**: [Contrib contnr3]The doctorate was then considered a work of maturity. I do not think that this was a happy vision, because there were some who took the teacher’s chair for twenty years and gave up finishing their doctorate. For others, having the doctorate lasted for their entire life, you know? Now, Ph.D. is a first attempt to start researching on a topic, to get a minimum of experience and assess the possibilities, capabilities and perspectives of a person. To not run late on the matter. A French mathematician, the friend of a Romanian mathematician, said that he had his doctorate quickly and the project's first sentence said this: “The main purpose of my work is to obtain the doctorate in mathematics.” It was a kind of statement of his, that he wanted to carry out research and that the doctorate was a first step; and I agree with that.

As it regards Piaget, genetic epistemology gave me a way out from the conventional dialectical materialism. Back then, in 1967, I was teaching a course called Theory of knowledge. I was a lecturer. Then I read Piaget, “Biologie et connaissance” convinced me. Piaget attracted me through the manner in which he stated that in the middle of the theory of knowledge there was the issue of the relationship between subject and object in building knowledge, whilst believing that it was not speculative, but based on clear research of genetic epistemology. Of course, I exaggerated the importance that I gave it then, but it was a way I could discuss something more professional at the time. Then Piaget came to Romania in 1971 because he received a Doctor Honoris Causa - and then I met him, after which I went to him with a von Humboldt scholarship. Nevertheless, I was already beginning to get far from genetic epistemology, as in Germany I had gotten in contact with analytic philosophy. It was a fleeting episode.

**EJOP**: We know that Americans are working with Major-Minor axes when it comes to offering expertise, which would provide premises for imminent professional skills, which brings a high level of performance that ultimately meets market requirements. Here, schools do not only become desirable, but they reconfigure their programs from a strictly mercantile perspective. Because of the recession, Greeks have recently wanted to take out organizational philosophy and psychology from the University of Athens. What are your hopes for the next century philosophy? Since the young philosopher does not have a domain in which to work, do you think that they can find a solution in interdisciplinarity, or would they better abandon philosophy?

**Mircea Flonta**: Many questions, I will try to answer, though. Philosophy today prepares mainly teachers of philosophy, although there are more graduates than education could absorb today. The usual destiny is the following: secondary education. Before, it was more room, with a single college in Bucharest and two departments at the faculties of History in Iasi and Cluj. Now things have changed. A graduate of philosophy can be used in a huge variety of activities, provided they think well independently, and speak and write well. In these conditions, you can be employed in any business! Same with any psychology graduate with similar characteristics. It is only that most graduates of philosophy of the last 15 years do not satisfy this condition. It could be wrong, but philosophy is now the path of young people who just come to have a degree, and I think it is easier than elsewhere. On the other hand, universities need students and there is competition to attract students, hence the decisive factor of lowering requirements. There is no other way. We do not have the American situation, where someone is impressed that you have a doctorate in philosophy at Harvard or at CUNY, etc. Here, grades are not representative and the faculty is not able to sort students. Therefore, there are increasingly fewer students, especially in theoretical philosophy, because it is considered more difficult - when telling stories about philosophical history seems easier and topics like politics and morals are more familiar than the logical themes of philosophy of language, philosophy of mind, etc. It is difficult to make a selection and there is no coverage for diplomas. My opinion is that both master and doctorate titles are given for the same qualifications to persons whose performances are very different, unacceptable different. I believe that things cannot continue in the current manner.

I think that the future of philosophy at the university would be other. Maybe a Department of Philosophy for the University, not for college. We give every student the opportunity to take a course in philosophy, or maybe two or three, to work and to obtain a degree, then those with a particular interest – a PhD in philosophy. Therefore, there are always students. I think intellectually active people, interested in ideas, in the company of such department and teachers with a professional attitude, who discuss topics of interest for good minds, can capitalize on opportunities and would take a bit of philosophy. My wife works with neuroscience and different projects in collaboration with universities in Europe, and some years ago, she has brought us a colleague in Hamburg. I did not know him, during our conversation, he asked me what I do for a living and I said philosophy. A, he says, I graduated from Heidelberg University (strong in biology and medicine) and I remember that I went to all of Gadamer’s classes. Gadamer was a professor at Heidelberg, i.e., if you have a modicum of pretention, you go there to see what people are talking about, right? It is as if you go to a show: they say there is a new play in town, right? People go; they should. This is how I see the future. However, it also takes another attitude from teachers from other faculties regarding their disciplines’ fundamental issues, which inevitably fall in connection with philosophy. This is what a true university professor should be like, one who can give students guidance.

I also have something more. Wittgenstein did not have any appreciation for philosophy as a profession; instead, he considered that every man who thinks with his mind must dabble in philosophy because philosophy is an exercise of the mind. Even if this exercise is not a routine, reaching towards philosophy is beneficial in key moments. Many believe that it is a matter of erudition – what you know about Plato, about Toma, about Descartes, Kant, etc. - but that is something that interests few people, and only esoterically. Instead, this thing, what advantage we have regarding our contact with representative philosophers to help us think better on what interests us as people active in a certain field, and as people who think about life and our relationships with others, is a work that seems to be important.

**EJOP**: Do you think the over-specialization in philosophy damages the philosophical work of an author?

**Mircea Flonta**: This is often the condition for a person with possibilities of performance in research. It is also compulsory. Let me tell you a little story. Einstein, from the 1920s until his death, worked all the time, intensely, but unlike the period before, he published almost nothing. He gave the following explanation - because I was only interested in matters of fundamentals and attempts to find a new path, I worked a lot with few results. Well, the common person cannot afford that. He or she does not have that capital. With capital, you can engage in some intellectually adventurous undertakings, meaning that they have a higher risk rate. The researcher cannot usually do that. He or she specializes, knows very well the literature of that narrow field, works and thinks by himself or herself, waiting for a result that falls like in a mosaic of science, like a pebble right where it should fit. It is what Thomas Kuhn calls “normal science” and it is studied by most scholars, most of their lives. It is somewhat mandatory. Of course, it is possible to obtain good quotations, academic positions, honors, influence, prestige, etc., but usually, this does not cause things to be remembered. Those who managed the opposite escaped the normal and narrow specialization of research, with a few risks here and there, because such work does not favour undertakings of large scale, while those who were accustomed to 30-40 years like this will continue so for the rest of their lives. Of course, they are very important as well.

In philosophy, there are three major examples – the specialized researcher who can publish quality works anywhere, and there are few here. The second figure is the writer of philosophy, who looks down on it and writes for a wide audience. The last one is the teacher who can afford to have research interests in a broader horizon, because he or she does not fall under pressure like the one who only deals with research. It is good to have room for all. We usually exaggerate with the criticism of one direction or another. Moreover, here, there is an editorial performance style, we do not have the peer-review system, the impartial referees, the conscientious, the ones who do not know who the author is, who have standards, read carefully, write a comprehensive report – we do not have that. From this point of view, we hold a peripheral position, we not master English as natives, plus many other aspects resulting from here - we are not in relationships with people of important centres, etc. Yet, not only research should be encouraged, as in Romanian will appear only what is regarded as worthless, because it cannot occur in English! The other danger is the formally executed work, books written to qualify for various posts, usually compilations, and so on.

We need a competent publisher, willing to invest time. We do not have the willingness to look seriously at things. You know how a journal rejecting an article looks like: it is a little piece of work, which is not paid for, perhaps only with prestige, according to the magazine and its board. Things are done very conscientiously. It is considered a second-class activity, like our situation. See doctoral papers. Do we have the guarantee that the reviewer has read everything with all the attention necessary and that the reviewer is one of the people who knows very well the literature of that particular theme? I have reasons to doubt.

**EJOP**: Do you think that the desire to write becomes more intense with age?

**Mircea Flonta**: With age, something not desirable can happen, but it does happen very often. As the years pass, you become more independent, look things up and you tend to think you have to say things more important than before, but you lose sight of the fact that regarding inventiveness and creativity, you do not gain points over the years. Therefore, many people are too active from a certain age, especially in the humanities, and if they hold a position, they cannot find anyone to say to them, with great respect, consideration and affection: “please know that you have written better before”. It is good to know where you can go once the years pass by. I read some things that I wrote when I was about 50-60 years old and I know I would not be able to do this thing, because I would not be able to hold and lead such a high number of threads in the analysis. There are disadvantages.

**EJOP**: Dear Professor, we thank you so much for the interview and we wish you the best.

